# Traumatic Intramural Jejunal Hematoma: A Case Report and Review of Literature

**DOI:** 10.7759/cureus.31458

**Published:** 2022-11-13

**Authors:** Shishir Kumar, Shivraj Chauhan, Kumar Diwakar

**Affiliations:** 1 Department of Surgery, Tata Main Hospital, Jamshedpur, IND; 2 Department of General Surgery, Tata Main Hospital, Jamshedpur, IND; 3 Department of Pediatrics, Tata Main Hospital, Jamshedpur, IND

**Keywords:** jejunal intramural hematoma, bilious vomiting, acute abdomen, intestinal obstruction, blunt abdominal trauma

## Abstract

We report a case of a jejunal hematoma in a six-year-old boy with an antecedent history of trauma. The development of duodenal hematoma post blunt abdominal trauma is well known; however, the jejunal hematoma is very rare. A six-year-old boy was brought to Tata Main Hospital (TMH), Jamshedpur, with pain abdomen, associated with a history of trauma to the abdomen. There were no signs of peritonitis on clinical examination. Initial ultrasonology revealed mild free fluid in the abdomen. CT abdomen was suggestive of intramural hematoma in the jejunum. Exploratory laparotomy findings were in concordance with CT abdomen findings. Resection-anastomosis of the jejunum was done. The patient was discharged uneventfully on postoperative day 7.

Blunt trauma to the abdomen is the principal cause of jejunal hematoma. The trauma in the majority of cases is trivial and usually the patients present late. The symptoms range from mild abdominal pain to intestinal obstruction with acute abdomen. A trial of conservative management is justifiable in a stable patient. If no clinical improvement is seen, surgical intervention is indicated. Surgical exploration was necessary because of signs and symptoms of intestinal obstruction due to jejunal hematoma. In pediatric cases, loss of functional bowel length and sudden decompensation due to expanding hematoma favor early exploration.

## Introduction

Intra-abdominal bowel and solid organ injuries after blunt abdominal trauma are significant causes of mortality/morbidity in the pediatric age group. Hematoma is predominantly seen in solid intra-abdominal organs and fixed and relatively immobile segments of the gut, like the duodenum [1]. Perforation is predominantly observed in the mobile part of the intestinal tract [2]. We report a rare case of intestinal obstruction associated with jejunal intramural hematoma post blunt abdominal trauma in a child managed in our institute. Conservative therapy was initially started but it failed and eventually it required surgical intervention.

## Case presentation

A six-year-old boy presented to us with a complaint of pain abdomen for seven days. There was a history of injury from the bicycle handlebar about 10 days ago. Initially, the boy was managed with enteral analgesics at a local hospital on an outpatient basis. The boy was tolerating oral feeds, with normal bowel and bladder movements. However, the boy was referred to us due to persistent pain.

Apart from the mild pallor, the general clinical examination was unremarkable with stable vital parameters. The abdominal examination was normal and there was no evidence of focal tenderness or lump. Ultrasonography abdomen was suggestive of mild free fluid. Hemoglobin level at presentation was 10.5 g/dl, total leucocyte counts 9,200/cmm, with 73% neutrophils. Platelets were adequate with counts of 185,000/cmm. INR was 1.1, while APTT was 27s (normal range: 23-33.4).

**Figure 1:**
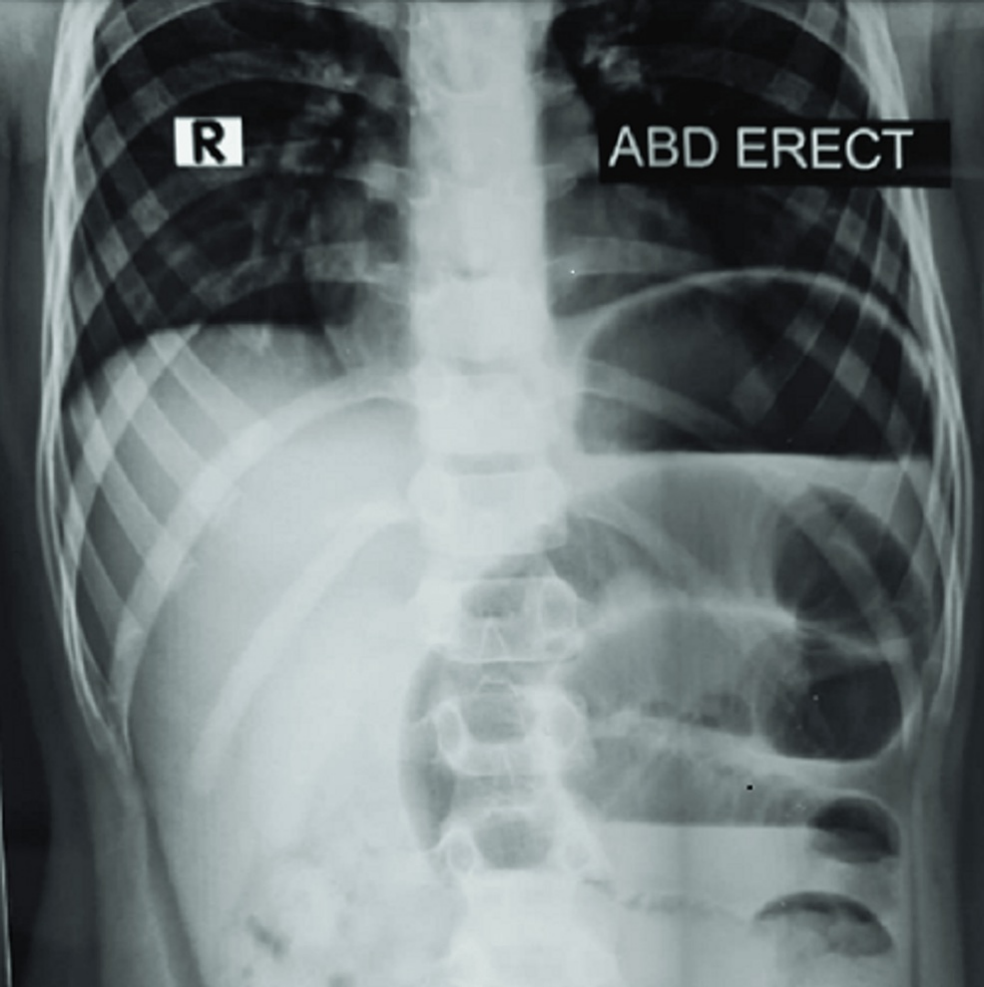
X-ray abdomen with features of intestinal obstruction

**Figure 2:**
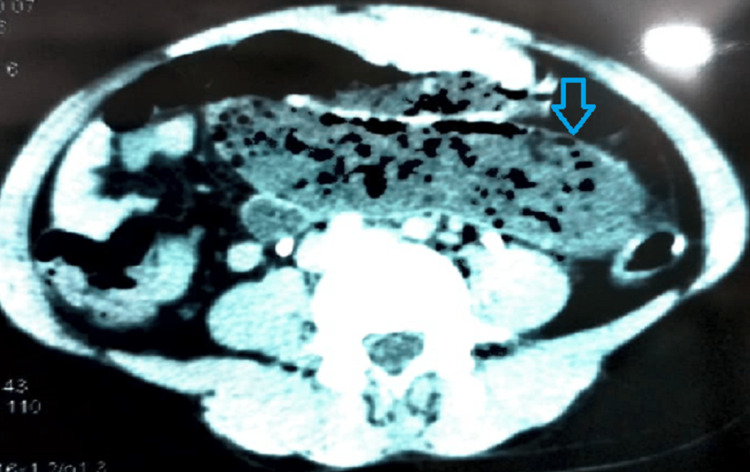
Sagittal section of abdomen showing hematoma in jejunum (blue arrow)

**Figure 3:**
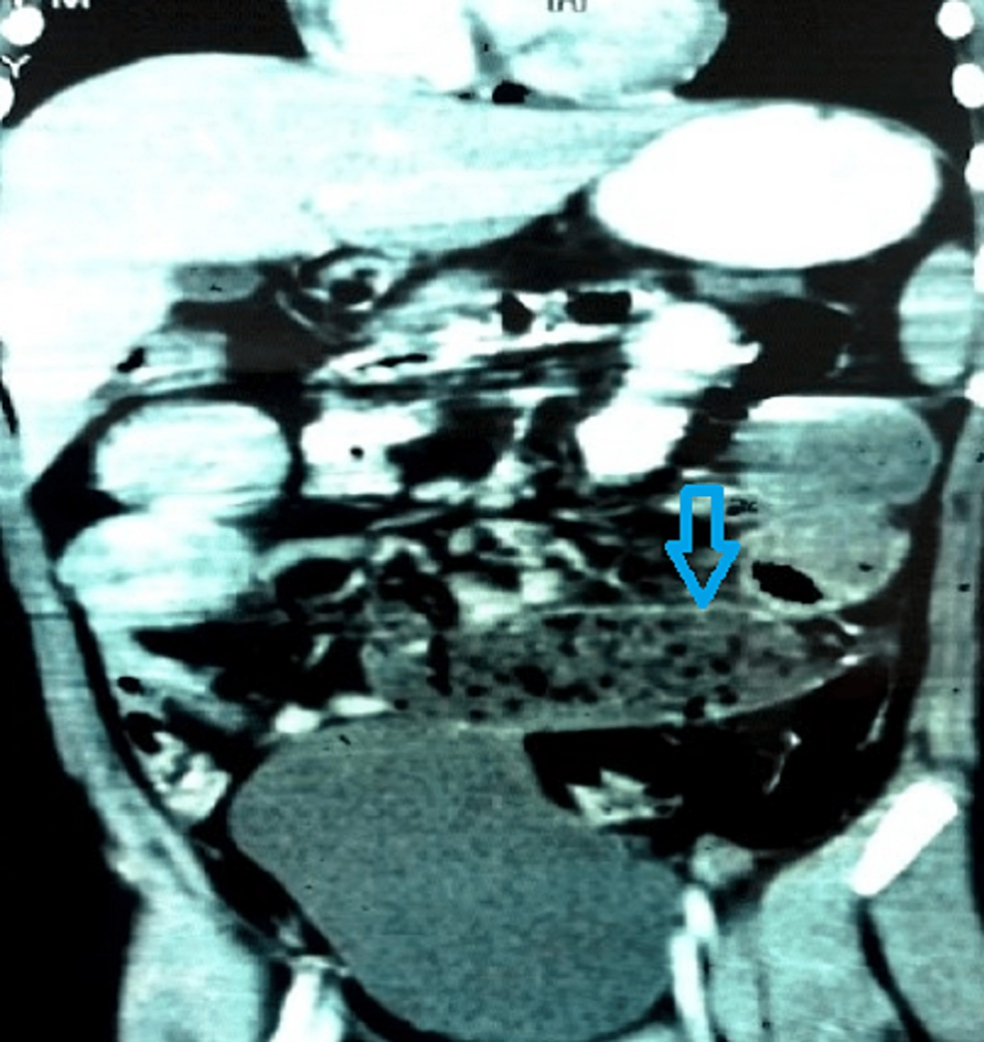
Hematoma in jejunum in coronal section of abdomen (blue arrow)

**Figure 4:**
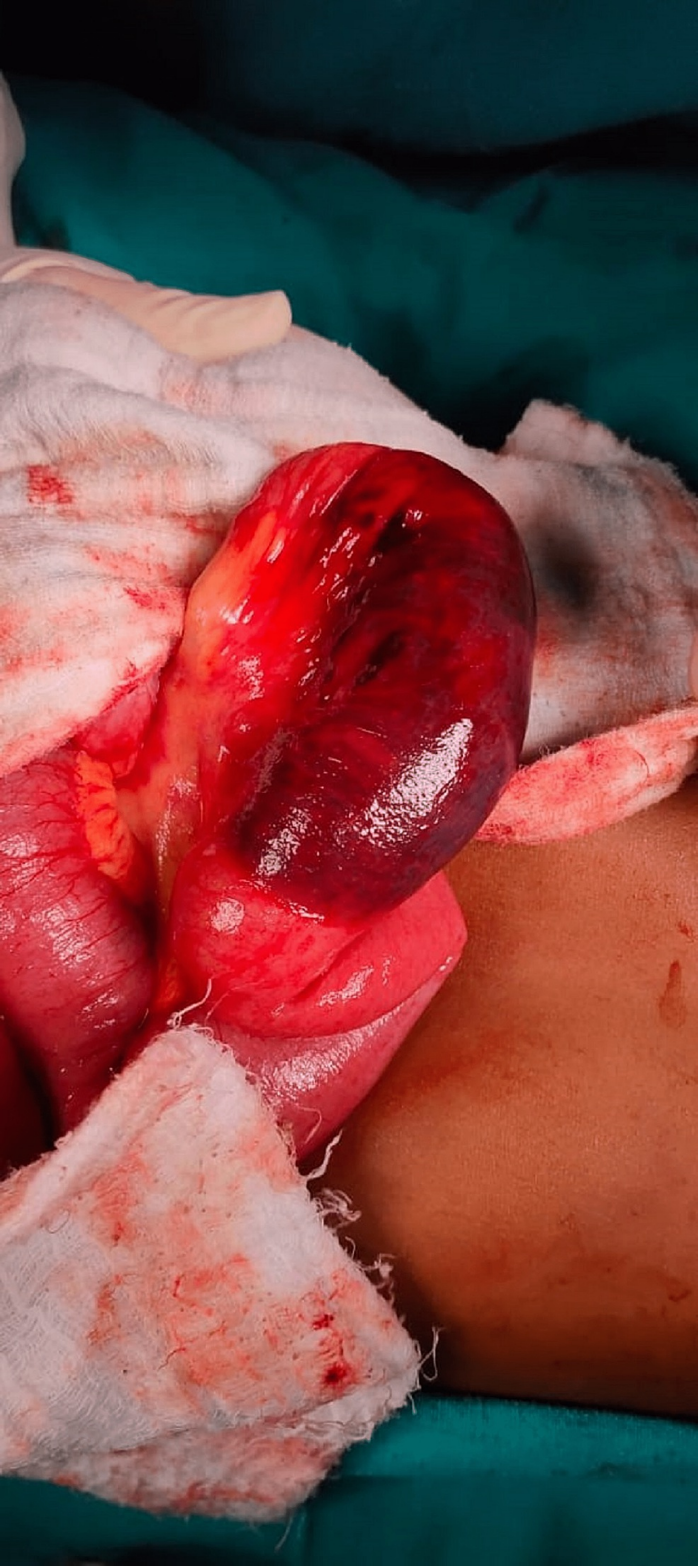
Intraoperative picture showing intramural hematoma in loop of jejunum

**Figure 5:**
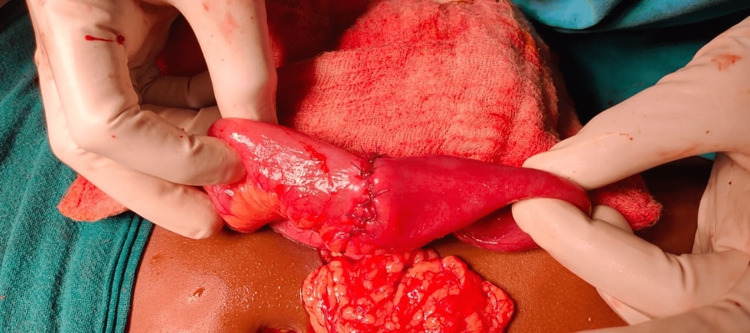
Jejuno-jejunal anastomosis post resection of jejunal hematoma

On day 1 of admission, the boy had an episode of bilious vomiting. X-ray abdomen showed multiple air-fluid levels (Figure 1). Contrast-enhanced CT (CECT) scan of the abdomen showed a hematoma in the jejunum, of dimension 8.6x2.8 cm (Figures 2, 3). The normal coagulation profile ruled out any association with bleeding diathesis. Considering the clinical features suggestive of intestinal obstruction with CECT finding of jejunal hematoma, the decision to perform laparotomy was taken. Informed written consent was obtained from parents. Exploratory laparotomy revealed mild hemoperitoneum, with hematoma involving a mid-jejunal segment of length 8 cm (Figure 4). Bowel loops distal to the hematoma collapsed. No luminal perforation was noticed. The jejunal segment with hematoma was resected, and intestinal continuity was restored by jejuno-jejunal anastomosis (Figure 5). Oral feeds were started on postoperative day (POD) 3, with full feeds achieved by POD 5. The boy was discharged uneventfully on POD 7.

## Discussion

Blunt injuries to the abdomen are a significant cause of morbidity and mortality in children. The most common mode of presentation is perforation in hollow viscus and laceration, hematoma in solid organs. There may be a significant delay between the onset of injury and the onset of symptoms as most abdominal trauma may be mild or trivial, that go unnoticed by the patient or the family. In our case, the boy presented to us after 10 days of abdominal trauma with a handlebar. Although he continued to experience pain abdomen, it did not affect his daily activities. The history of trauma with a handlebar could be elicited only after detailed questioning as the parents assumed that the trauma due to the handlebar was inconsequential.

In a solid organ injury following blunt abdominal trauma, laceration and hematoma are usually seen while hollow viscus presents with perforation. Hematoma is frequently seen in the retroperitoneal segment of the duodenum. Bleeding diathesis needs to be ruled out in children presenting with hematoma [3]. In contrast to adults, children are vulnerable to developing jejunal hematoma on account of the smaller anteroposterior depth of the abdominal cavity and underdeveloped abdominal musculature that prevent them to endure the traumatic insult [4]. In our case, the coagulation profiles were normal. Hematoma progressively distends and grows on account of lingering bleeding and absorption of fluid secondary to elevated osmolarity brought about by the protein products of hemoglobin disintegration. This may explain the delay in obstructive features observed in an intramural jejunal hematoma [5]. The diagnostic modality of choice in such cases is CT abdomen, which may show circumferential thickening, intramural hyperdensity, and luminal narrowing [6]. Surgical intervention is necessitated if the patient develops intestinal obstruction; however, the management options remain undefined in a hemodynamically stable child without features of intestinal obstruction. In our case, the decision to intervene surgically was based on features of intestinal obstruction. Surgical management may be deferred in a hemodynamically stable child if hematoma does not lead to obstructive features. However, the inherent peril, in this approach is the risk of expansion of hematoma, leading to the loss of a functional segment of the jejunum. The natural history of healing by fibrosis may result in a stiff bowel wall or stricture following conservative non-surgical management [7]. Hematoma involving a large segment of the bowel and expanding hematoma thus may be considered as indications of surgical intervention, especially in the pediatric age group, where the loss of functional bowel length, either immediate or in the future due to fibrosis of hematoma is a major concern. Image-guided aspiration of hematoma has also been reported, but that may not be able to evacuate the entire hematoma leaving behind residual hematoma [8]. Also, the condition of underlying mucosa cannot be observed in such cases, thus necessitating additional surgical procedures, despite the successful evacuation of the hematoma [9]. An additional concern in such procedures is conversion of a sterile hematoma to an infected one. In our case, the hematoma involved 8 cm of the length of the mid jejunum, causing intestinal obstruction, thus requiring surgical intervention.

## Conclusions

Seemingly inconsequential blunt abdominal trauma may lead to intramural jejunal hematoma in children, and it may result in intestinal obstruction after some time lag between injury and presentation. Surgical intervention is the preferred approach in a child who develops intestinal obstruction; however, there is a risk of loss of functional bowel segment and decompensation due to expanding hematoma in the conservative approach.
